# Recent topics on the scope of physiological anthropology

**DOI:** 10.1186/1880-6805-32-25

**Published:** 2013-12-27

**Authors:** Akira Yasukouchi

**Affiliations:** 1 Department of Physiological Anthropology, Faculty of Design, Kyushu University, 4-9-1, Shiobaru, Minami-ku, Fukuoka 815-8540, Japan

## 

Two years have passed since the *Journal of Physiological Anthropology* (*JPA*) adopted an open-access policy. The Editorial issued in 2012 provided an overview of the scope of the *JPA*[1] and reviewed the biological adaptations accomplished by *Homo sapiens* during the hunter-gatherer period, which spans most of human history, against the speed at which mankind has created artificial environments through the development of new technologies. In light of this, attention must be paid to how different the environmental pressures of today are compared with those of the past and what sort of impact this difference has on human adaptability. The discipline of physiological anthropology focuses on assessing exactly this human adaptability to the environment.

The diversity and mechanisms of environmental adaptability, including techno-adaptability, are investigated from the perspective of physiological polytypism, whole-body coordination and functional potentiality, three major keywords in physiological anthropology [2]. Based on these keywords and the scope of the *JPA*, this article reviews selected studies published in the *JPA* in 2012.

The major adaptation that enabled *Homo sapiens* to establish its unique abilities is bipedalism. This form of locomotion allowed for longer migration distances and improved visibility through providing a higher eye level, making hunting and gathering easier. However, an upright posture comes with the risks of having to increase blood pressure to pump blood even higher to the brain while venous return to the heart, which is situated relatively high within the body, tends to decrease against gravitational stress. Compared with quadrupeds, uprightness is a more challenging gravity-defying posture because high blood pressure must be maintained during any postural change. This is achieved via orthostatic cardiovascular responses, which are regulated by the autonomic nervous system and endocrine system as highly advanced coordinating reactions. However, today’s more sedentary lifestyle, due largely to modern transportation and office-based work, is thought to be weakening our orthostatic tolerance. Consequently, even in the field of physiological anthropology, many studies have investigated circulatory dynamics [3,4] and the orthostatic responses [5,6] in relation to postural change.

In a recent *JPA* article by Ishibashi *et al*. [7], the authors proposed a novel method to evaluate orthostatic cardiovascular responses by using lower body negative pressure [7]. Compared with the conventional method, this method utilizes sinusoidal negative pressure to accurately simulate the blood shift to the lower body. By measuring the effect of the constant load component during oscillatory lower body negative pressure, it is possible to elucidate the frequency characteristics of each function involved in the regulation of orthostatic responses and to visualize temporal changes in the whole-body coordination of blood pressure regulation. This study falls within the scope of the *JPA* since the method can be used to investigate physiological polytypism in the relation between today’s living-related environmental factors and orthostatic tolerance in humans.

Physiological anthropologists began the full-scale study of environmental adaptation in the second half of the 20th century [8,9]. In particular, the study of adaptation to thermal environments has a long history in Japan, as well as in the United States [10-12]. The International Biological Program (IBP) launched in 1964 was a large field study involving researchers from many countries, which was set up to investigate diverse environmental adaptations seen from an ecological point of view. In the late 1960s, Japan also started to investigate hypobaric and thermal adaptabilities using climate chambers in experimental studies.

Some studies have been performed on thermal adaptability when reviewed in light of ‘functional potentiality’, one of the keywords of physiological anthropology. By defining heat tolerance as the ability to maintain body temperature under heat stress, these studies showed that heat tolerance varies among the residents of the United States, Europe, Africa and Asia [13]. Even among Asian populations, the ability to maintain body temperature differs between Japanese residing mainly in the temperate zone and Malaysians acclimatized to a tropical zone. Moreover, heat tolerance has been shown to improve in Malaysians who repeatedly perform physical work in a hot environment [14], demonstrating that even the inhabitants of tropical areas have sufficient functional potentiality remaining in the thermoregulatory mechanism. However, the functional potentiality of residents in tropical Asian countries is modified once they move into a temperate zone. In a comparison of Japanese men and south-east Asian men residing in Japan, Wijayanto *et al*. [15] revealed that south-east Asian men who had resided in Japan for a long time had an earlier sweating onset time and higher sweat rate than south-east Asian men who had resided in Japan for a shorter time, demonstrating the decay of acclimatization in the sweating response [15]. From the perspective of physiological anthropology, this study is interesting in that high heat tolerance acquired through long-term heat acclimatization to a tropical is decayed (an augmentation of functional potentiality) by residing in a temperate climate. In other words, the physiological polytypism observed among different communities is plastic to climatic conditions.

It is known that adaptability to cold environments also exhibits physiological polytypism. For example, a previous study of physiological polytypism as a coordinated response to cold exposure showed that the Inuit people responded with an elevated metabolism, whereas the highland Quechua population of the Andes responded with increased thermal insulation. However, to fully understand the different phenotypic expressions, it is necessary to investigate environmental and nutritional factors; for example, whether consuming a high-calorie diet makes it possible to maintain an elevated metabolism in a given environment. Similarly, it is possible that in the study by Wijayanto *et al*. [15] environmental factors had more profound effects on heat adaptability than genetic factors. At present, it is unclear if and how genetic factors affect functional adaptability to the environment, and this is the current focus of study in physiological anthropology. Despite the human genome having been sequenced [16], physiological research on genetic adaptations has been delayed, partly because a relatively short time has elapsed since the official release of the International HapMap database [17,18] and because techniques and technologies are not yet sufficient to identify the link between functional phenotype and genes or genotype.

Against this background, the *JPA* published an interesting article [19] on the involvement of mitochondrial DNA (mtDNA) polymorphism in cold adaptation from the viewpoint of human migration. The authors focused on mtDNA haplogroup D, the predominant haplogroup among Japanese, and investigated the association between physiological polytypism and seasonal variation in thermoregulation against a cold environment to reveal the involvement of mtDNA polymorphism in cold adaptation. This kind of experimental study often has a limited number of subjects and additional studies are needed before we can come to any conclusion on the influence of genetic factors. Nonetheless, the study is interesting in that thermoregulation against cold stress, especially in summer, was clearly different between the haplotype D and non-haplotype D groups. This study warrants attention because it is the first physiological anthropological study of its kind to demonstrate objectively the relation between physiological polytypisms and genetic factors.

Here we have briefly introduced selected studies on physiological polytypism from the perspective of whole-body coordination and functional potentiality, both keywords in physiological anthropology, with the aim of helping *JPA* readers to understand the research aims and scope of the journal. It should be noted, however, that the *JPA* also publishes a large number of articles whose content has no direct association with these key concepts: we also welcome articles on the morphological and physiological characteristics of populations, in terms of age, sex and different regions, due to the variability of environmental adaptations in people today and those of the past. We look forward to receiving articles on various approaches for investigating environmental adaptability in mankind.
